# Advancing Clinical Practice: The Potential of Multimodal Technology in Modern Medicine

**DOI:** 10.3390/jcm13206246

**Published:** 2024-10-19

**Authors:** Yaara Artsi, Vera Sorin, Benjamin S. Glicksberg, Girish N. Nadkarni, Eyal Klang

**Affiliations:** 1 Azrieli Faculty of Medicine, Bar-Ilan University, Zefat 1311502, Israel; 2 Department of Radiology, Mayo Clinic, Rochester, MN 55905, USA; verasrn@gmail.com; 3 Division of Data-Driven and Digital Medicine (D3M), Icahn School of Medicine at Mount Sinai, New York, NY 10029, USA; ben.glicksberg@gmail.com (B.S.G.); girish.nadkarni@mountsinai.org (G.N.N.); eyalkla@hotmail.com (E.K.); 4 The Charles Bronfman Institute of Personalized Medicine, Icahn School of Medicine at Mount Sinai, New York, NY 10029, USA

**Keywords:** artificial intelligence, multimodal, clinical practice, technology, health-care

## Abstract

Multimodal technology is poised to revolutionize clinical practice by integrating artificial intelligence with traditional diagnostic modalities. This evolution traces its roots from Hippocrates’ humoral theory to the use of sophisticated AI-driven platforms that synthesize data across multiple sensory channels. The interplay between historical medical practices and modern technology challenges conventional patient–clinician interactions and redefines diagnostic accuracy. Highlighting applications from neurology to radiology, the potential of multimodal technology emerges, suggesting a future where AI not only supports but enhances human sensory inputs in medical diagnostics. This shift invites the medical community to navigate the ethical, practical, and technological changes reshaping the landscape of clinical medicine.

## 1. Introduction

Medicine is a multimodal discipline. Hippocrates of Kos (460-377 Before Common Era, BCE), who is considered the father of modern medicine, theorized that the body consisted of four fluids or ‘humors’, black bile, yellow bile, phlegm, and blood, requiring the basic sense of vision [1]. In 1816, René Théophile Hyacinthe Laënnec invented the stethoscope and, 3 years later, published his revolutionary masterpiece “De L’Auscultation Médiate”. This tool allowed him to hear, interpret, and document heart and lung sounds [2]. Naturally, tactile touch is considered the basis for human–patient communication and is a fundamental part of physical examination and clinical practice [3]. Smell was also used for centuries as a diagnostic tool in the practice of medicine, be it for recognizing gas gangrene on the battlefield or diabetic ketoacidosis in the emergency room [4]. Even the sense of taste was applied; for example, the sweet taste of diabetic urine, which is described in ancient Indian texts and noted by Avicenna (980–1037) and Morgagni (1635–1683), was attributed to the passage of absorbed water and nutrients unchanged into the urine [5].

We use our senses to interpret the world around us, and this fundamental process is mirrored in medicine. Without it, there could be no possibility to diagnose, treat, and communicate with patients. It is almost impossible to imagine a world where the human component in the medical profession could be replicated or supplanted by artificial systems—until now.

In recent years, clinical practice has been transformed by significant advancements in technology, research, and patient care methodologies [6]. In the early days, medical practice relied heavily on physical examination, basic human senses, and rudimentary diagnostic tools. The advent of advanced imaging technologies like MRI and CT scans, along with the introduction of minimally invasive surgical techniques, significantly enhanced diagnostic accuracy and treatment precision [7]. The digital revolution in the late 20th and early 21st centuries brought about electronic health records (EHRs), which streamlined patient data management and improved coordination among healthcare providers [8].

Telemedicine and mobile health technologies have also expanded access to care, allowing patients to receive medical consultations and monitoring remotely [9]. Recently, the integration of artificial intelligence (AI), machine learning (ML), and big data analytics has taken the healthcare community and medical profession by storm, with its potential transformative power on current clinical practice.

Multimodal technology encompasses the integration of multiple forms of data and sensory inputs to process information. By analyzing information from different sources, this technology provides a more comprehensive and accurate understanding of complex situations or problems, potentially enabling more accurate diagnoses in medicine.

The aim of this perspective is to critically explore and provide a comprehensive overview of the integration of multimodal AI technologies in modern clinical practice, examining both their potential and limitations, and to analyze the key components of AI which facilitate this ability.

Moreover, this perspective aims to offer a balanced analysis by not only highlighting the promising applications of AI across various medical specialties but also discussing the significant challenges that impede its full integration.

## 2. Fundamental Concepts

### Multimodal Technology and Its Components

Several components of multimodal technology are what makes its implementation in clinical practice interesting to research.

Data diversity: the collection and analysis of diverse types of data, such as text, images, audio, video, sensor readings, and diverse types of biomedical information, allow for a richer and more nuanced interpretation of information.

Integration and fusion of different data types are central to multimodal technology. This involves combining data in a way that capitalizes on the strengths of each modality, providing a more holistic view than any single data type could offer.

Machine learning and artificial intelligence are employed to process and analyze multimodal data. Machine learning models can identify patterns and correlations across different data types, enabling more accurate predictions and insights.

Interoperability ensures that data from different sources can be integrated smoothly. For multimodal systems to function effectively, they must be able to communicate and work seamlessly with various devices and platforms.

User interaction via multiple sensory channels, such as visual, auditory, and tactile interfaces, enhances user engagement and accessibility, allowing for more intuitive and efficient interactions.

Artificial intelligence (AI): Artificial intelligence, or AI, is a branch of computer science focused on creating machines or software that can perform tasks that usually require human intelligence. These tasks include things like understanding language, recognizing patterns, solving problems, learning from experience, and making decisions [10].

Machine learning: Machine learning is a subset of artificial intelligence (AI) that enables computers to learn and improve from experience without being explicitly programmed for every task. In a way, it is teaching a computer how to do something by giving it lots of examples, rather than telling it exactly what to do step by step.

The key idea is that instead of writing a specific set of rules for the computer to follow, it will generate its own rules by analyzing the data. This makes machine learning very powerful due to the fact that it can adapt to new situations and improve its performance as it obtains more data or experience.

Deep learning: Deep learning is a type of machine learning that tries to mimic how the human brain works in processing information and making decisions. It is called “deep” because it uses many layers of processing, with each one refining the information a little more, just like how the human brain layers different pieces of information to understand something complex [11].

Imagine trying to recognize a face in a crowd. First, the brain might pick out basic features like shapes and colors. Then, it might identify more specific features like eyes, a nose, and a mouth. Finally, it puts everything together to recognize whether it is a friendly face. Deep learning works in a similar way, with each layer of processing in the computer handling different levels of detail.

In practical terms, deep learning drives many of the latest technological advances, like autonomous cars (helping them understand the road and objects around them). Deep learning also enables advanced natural language processing systems, which allow speech recognition applications, voice assistants (like Siri and Alexa, that understand and generate human-like responses), and chatbots such as ChatGPT. It is especially useful for tasks that involve complex data, like images, sounds, and natural language.

Artificial neural networks: Deep learning models are often built using structures called artificial neural networks, which are inspired by the way neurons in the human brain connect and work together. These networks can learn to recognize patterns and make decisions in ways that are often more accurate than traditional machine learning methods, especially when there are a lot of data to learn from. Artificial neural networks comprise multiple layers of nodes, known as artificial neurons. Each neuron simulates a single logistic regression unit, processing inputs to produce an output through an activation function. This function determines whether the neuron fires, akin to a binary decision in logistic regression. The inputs to each neuron are weighted, emphasizing the importance of some over others. Through training, these weights adjust to minimize errors in predictions, enhancing the model’s accuracy over time. Networks with many layers, called “deep” are capable of abstracting data at multiple levels, recognizing intricate patterns in large datasets [11].

Transformers: Transformers are a type of deep learning model that have become incredibly powerful in processing and understanding language. It is a similar process to reading a sentence; as the brain goes through each word, it keeps track of the context—the meaning of the words before and after the current word. Transformers work in a similar way but on a much larger scale.

At the heart of transformers is a mechanism called “attention” [12,13]. This allows the model to focus on different parts of the input (like words in a sentence) and understand how they relate to each other, even if they are far apart in the sentence. For example, suppose the task is to analyze and determine whether a patient has a certain condition, such as diabetes, based on an EHR record. The transformer model can “pay attention” to key pieces of information scattered throughout the input data, such as the following:

- Blood sugar levels noted in laboratory results.
- Symptoms such as frequent urination or excessive thirst documented in clinical notes.
- Medication history related to blood sugar management.
- Past diagnoses that might indicate risk factors, such as obesity or hypertension, or associated conditions such as diabetic retinopathy.

The attention mechanism allows the model to weigh the importance of each piece of information in the EHR relative to the task. This innovation differs from traditional feature importance as it is context-specific, meaning attention is dynamically shifted with each input. It might give more “attention” to recent lab results showing elevated blood sugar levels, while also considering relevant symptoms mentioned in the patient’s history. Even if these details are spread across different parts of the record, the model can identify and prioritize them, helping to make a more accurate diagnosis.

Transformers are used in many applications, especially in language processing tasks like translation, summarization, and text generation. They are the technology behind some of the most advanced AI models today, such as large language models (LLMs) including the Generative pre-trained Transformer (GPT) family of models and Bidirectional Encoder Representations from Transformers (BERTs). These models are used for understanding and generating human-like text [14]. Because of their ability to handle large amounts of data and understand complex language structures, transformers have revolutionized the field of natural language processing.

Transformers have also been used for image analysis. Using similar attention mechanisms as with natural language processing, the model can focus on specific areas within an image deemed “important”. For example, Azad et al. reviewed how Vision Transformers (ViTs) have advanced medical image analysis. ViTs outperform traditional convolutional neural networks (CNNs) in learning long-range dependencies and spatial correlations, making them highly effective for complex medical imaging tasks like classification, segmentation, and detection [15]. Some key challenges include the large computational resources required to train Vision Transformers and the need for substantial annotated medical datasets. While ViTs demonstrate remarkable improvements in handling diverse medical imaging modalities, their application is often constrained by the availability of high-quality data, which is crucial for precise medical diagnosis.

## 3. Leading Multimodal Tools and Platforms

New multimodal models emerging recently such as GPT4Vision (GPT4V), Gemini, and Whisper (Table 1) can interpret and generate not only text but also images, videos, and sound [16]. By using few-shot learning, the newer models are better at clearing ambiguity, understanding clinical context, understanding the expected format the response should take, and aiding in reasoning [17]. The use of diverse data types such as imaging, sound, genomics, biometrics, and EHR notes can create a comprehensive view of a patient’s health status.

## 4. Clinical Usage

Since their release, several studies have already tested the different multimodal capabilities in clinical settings. Zhu et al. performed a pilot study on ChatGPT-4V (Vision)’s ability to interpret radiological images [28]. They found that ChatGPT-4V achieved a diagnostic accuracy of 77.01% for USMLE-style questions, with an average score of 3.97 for treatment plans. By removing detailed patient history, the diagnostic accuracy dropped to 19.54% (*p* < 0.0001). Also, without detailed patient history, ChatGPT-4V could not specify the exact disease, but was able to provide diagnoses consistent with or very similar to the reference answers. Elyoseph et al. tested Chat-GPT 4’s ability in mentalization, interpreting human emotions from visual and textual data. They found that ChatGPT-4 proved its efficacy in the domain of visual mentalizing, aligning closely with human performance standards [29]. In clinical settings, multimodal technology can transform patient care by providing comprehensive diagnostics through imaging data integration [30,31,32] with other health metrics, such as lab results and patient history, achieving more accurate diagnoses.

Multimodal data enable healthcare providers to tailor treatments to individual patients based on a comprehensive analysis of various health factors, creating personalized treatment plans [33]. They can analyze collected real-time data using remote monitoring and wearable sensors, allowing for the continuous monitoring of patients’ conditions outside traditional clinical settings. Enhanced multimodal data interfaces, such as virtual reality or augmented reality, can improve patient understanding and involvement in their treatment plans, enhancing patient engagement [34]. The study by Vikram R explores the use of automated multimodal systems for the remote monitoring and assessment of neurological and mental health conditions [35]. It emphasizes the integration of various data types, such as speech, facial expressions, and cognitive performance captured through remote interactions. These systems utilize AI-driven analysis to detect and monitor health conditions, making it a critical tool in the telehealth ecosystem, especially for conditions like Parkinson’s and other neurodegenerative disorders. This study highlights the potential of such technologies to enhance early diagnosis, treatment, and ongoing monitoring, particularly in a post-COVID 19 pandemic world where remote healthcare is increasingly important.

Sorin et al. evaluated the capability of GPT-4’s multimodal version (GPT-4V) to analyze ophthalmology cases that involve both textual data and ocular images [36]. They provided GPT-4V with ophthalmological images, initially without clinical context, and later with clinical data for comparison. This study involved 40 patients with various ocular pathologies. Without clinical context, GPT-4V achieved correct diagnoses in 47.5% of cases, but the accuracy improved to 67.5% when the clinical context was provided. This study underscores the model’s capability to integrate multimodal data effectively, with significant promise for future advancements in AI-driven medical diagnostics.

Applying multimodal technology can also assist in treatment plans after hospitalization and throughout different treatment phases. The study by Indolfi et al. examines the use of artificial intelligence (AI) in managing allergies, particularly focusing on the transition of care from childhood to adulthood [37]. This research highlights AI’s potential in improving personalized allergy care through enhanced risk stratification, treatment optimization, and remote patient monitoring. AI can aid in the continuous tracking of a patient’s allergy progression and help healthcare providers make better-informed decisions during the transition phase. However, this study emphasizes that while AI is a valuable tool, it cannot replace the human elements of empathy and ethical judgment in patient care, making AI a complementary rather than standalone solution in allergy management.

Another study by Zhu et al. explored the impact of upgrading management processes in hospital operating departments using multimodal identification technology [38]. This research compared traditional surgical management practices with the new multimodal system in 4630 laparoscopic surgeries conducted over two years. They found that the multimodal system significantly improved various aspects of the operating department’s efficiency. Among the 251 cases in the control group, 198 cases were on time, and the rate of on-time operation was 78.9%. In the multimodal practice group, 229 cases were on time, and the rate of on-time operation was 92.3%, with a significant difference in the rate of on-time operation between the two groups. The waiting times for consecutive surgeries were compared between the two groups. The multimodal practice group had significantly shorter waiting times for consecutive surgeries relative to the traditional group. In addition, the mean operative time for all procedures was significantly shorter in the multimodal practice group relative to the traditional group. This multimodal approach enhanced the speed and efficiency of surgical procedures, improved material management, and increased patient satisfaction with their surgical experience. These improvements suggest that integrating advanced identification technologies into surgical management can lead to better outcomes both operationally and from the patient’s perspective.

Even when comparing multimodal to single modality technology, studies already show a clear advantage in favor of multimodality. Kwon et al. evaluated the use of deep learning to improve outcome prognoses for COVID-19 patients in the emergency department by combining initial radiographs with clinical variables [39]. The model trained on the chest radiograph severity score produced the following areas under the receiver operating characteristic curves (AUCs): 0.80 (95% CI: 0.73, 0.88) for the chest radiograph severity score, 0.76 (95% CI: 0.68, 0.84) for admission, 0.66 (95% CI: 0.56, 0.75) for intubation, and 0.59 (95% CI: 0.49, 0.69) for death. The model trained on clinical variables produced an AUC of 0.64 (95% CI: 0.55, 0.73) for intubation and an AUC of 0.59 (95% CI: 0.50, 0.68) for death. Combining chest radiography and clinical variables increased the AUC of intubation and death to 0.88 (95% CI: 0.79, 0.96) and 0.82 (95% CI: 0.72, 0.91), respectively. These results show that combining both data types improved the model’s ability to predict outcomes such as intubation and mortality, with significantly higher accuracy than using either radiographs or clinical variables alone. This demonstrates the potential of integrating multimodal data to enhance predictive accuracy in critical care settings. A different study by Mohsen et al. examined the integration of AI techniques to combine EHRs with medical imaging data [40]. This fusion aimed to enhance clinical decision making by providing a more comprehensive view of patient health.

The researchers conducted a detailed review of various AI methods used for this purpose, focusing on how different data types can be combined to improve diagnostic accuracy and patient outcomes. This study found that AI-driven multimodal fusion models, which combine EHRs and imaging data, generally outperform models that rely on a single data modality. The most common applications of these fusion models were in disease diagnosis and prediction, with early fusion techniques being the most widely used.

Further proof of the multimodal superiority to single data models is seen in the study by Lipkova et al. which explores the use of AI to combine various types of medical data, such as radiology, genomics, and pathology, for improved cancer diagnosis and treatment [41]. This research highlights that integrating these different data sources enhances predictive models for cancer outcomes and can even identify novel biomarkers. This study emphasizes that multimodal data fusion using AI outperforms single-modality approaches, leading to more personalized and accurate oncology care.

Multimodal data are not only used to advance oncologic care, but they can also be used to predict various gene mutations that can assist in tailored care. The following study by Shao et al. focuses on using AI and multimodal integration (MMI) to predict gene mutations, advancing precision oncology. By combining diverse data sources like medical imaging, genomics, and clinical data, AI models were developed to improve mutation status predictions, essential for tailoring personalized cancer treatments [42]. This study emphasizes that MMI-based AI models outperform single-modality approaches in predicting mutations and offer significant potential for more accurate diagnoses and treatment plans in oncology. These findings emphasize that by integrating diverse data sources, healthcare providers can gain deeper insights, leading to more personalized and effective treatments for patients.

In contrast, an important aspect of clinical care is mental health, as well as physical. The study by Alhuwaydi explores the growing role of artificial intelligence (AI) in mental healthcare [43]. The author provides insights into current trends and potential future directions in the narrative review, discussing how AI technologies, such as machine learning and predictive analytics, are being applied to improve screening, diagnosis, and treatment in mental health. These AI-driven tools help analyze vast datasets to predict patterns related to mental illnesses, offering individualized and preventive care.

However, the review also highlights challenges, such as the need for human empathy in mental healthcare. AI lacks human emotional intelligence, which may impact the quality of care, as mental health often requires compassionate, nuanced human interaction [44], as well as ethical concerns, due to the potential of AI systems to reinforce biases and exacerbate inequalities in mental health treatment [45]. Further challenges that were mentioned were data privacy issues and the sensitive nature of mental health data, which raises privacy risks, especially in AI-driven systems [46], and the cultural sensitivities involved in AI applications, especially in mental health, where cultural differences play a key role in shaping how individuals perceive health, illness, and treatment [47]. This study stresses the importance of collaboration between healthcare professionals and AI to ensure effective and ethical use of these technologies in mental healthcare. It also calls for more research and larger studies to fill existing gaps, especially regarding how AI can complement human-centered approaches in mental health.

A study conducted in a similar field also stresses the impact of AI on clinical care, both positive and potentially negative. The study by Ettman and Galea discusses the significant potential of AI to impact population mental health [48]. The authors propose three primary areas of influence. Advancement in mental healthcare: AI has the potential to improve the prevention, screening, and treatment of mental health disorders by enabling early detection, predicting risks, and offering digital interventions. AI tools could help identify high-risk populations and provide quicker, more accessible interventions, especially in underserved areas. Shifts in social and economic contexts: AI might reshape economic landscapes, possibly exacerbating mental health disparities by displacing jobs or increasing inequality. Alternatively, AI could create new opportunities, balancing its impact by offering new economic pathways. Policy and regulation: The authors stress the importance of developing policies that safeguard patient privacy and reduce algorithmic bias. Proper regulatory frameworks are essential to prevent AI from worsening existing mental health disparities and ensure ethical use in healthcare.

This study also emphasizes that while AI offers promise, there are significant risks associated with its adoption, such as privacy concerns and the potential for misuse in mental healthcare.

Theoretically, with the further advancement of this multimodal technology, multimodal data could replace many functions performed today by medical staff, like interpreting a video of a patient with respiratory distress and alerting the ED, diagnosing a disease based on the pathology image provided to the model, or performing personalized treatment plans for individual patients, based on risk factors and personal history. Multimodal data might also be able to delve into the world of psychiatric medicine, by their growing ability to interpret images expressing human emotions [29]. In a sense, multimodal tools could practically augment human senses with input from “machine senses”.

## 5. Medical Education

Another aspect of clinical practice is medical education. The utilization of AI technologies in medical education presents an intriguing opportunity to enhance learning and decision making by providing a new dimension of learning for students and clinicians. Additionally, medical students may benefit from using AI as a supplementary learning tool, as it can offer real-time feedback and enhance understanding of clinical reasoning through interactive learning.

The study by Kung et al. evaluated the performance of ChatGPT on the United States Medical Licensing Examination (USMLE) to assess its potential for AI-assisted medical education [49]. They tested ChatGPT across all three levels of the USMLE, which cover a wide range of medical topics, including basic sciences, clinical knowledge, and applied clinical skills. The AI model achieved an accuracy of approximately 60% in answering questions, which is near the passing threshold for the exam. However, limitations, such as difficulties with nuanced clinical reasoning and interpreting certain clinical scenarios, were noted.

While ChatGPT shows promise for assisting in medical education, particularly in helping students prepare for exams, reinforcing learning, or even generating medical exams for testing [50], it still lacks the ability to interpret the subtleties of human interaction, emotional intelligence, and ethical judgment, which remain crucial aspects of clinical practice that seasoned physicians bring to their decision-making process [51].

## 6. Limitations of AI

Despite its potential, the application of AI technology in medical clinical practice faces significant challenges that hinder its widespread adoption. One of the primary challenges is the “black box” nature of many AI algorithms, where the decision-making process is opaque, making it difficult for clinicians to trust or fully understand how a diagnosis or recommendation is generated [52]. This lack of transparency raises concerns about accountability, especially in cases where AI-driven errors occur, such as misdiagnoses or inappropriate treatment suggestions. Moreover, AI models often rely on large, high-quality datasets for training, and if these datasets are biased or unrepresentative, the models can perpetuate and even exacerbate healthcare disparities, particularly in underserved populations [53]. Another known limitation is a phenomenon known as “hallucination”. This occurs when generative AI misinterprets the given prompt in a wide variety of scenarios, resulting in outputs that lack logical consistency or in completely false information [54]. When relying on AI for quality diagnosing and medical recommendations, this phenomenon is unacceptable. Additionally, AI implementation requires significant financial investment, along with extensive training for healthcare staff, which poses a barrier for resource-limited healthcare facilities. Finally, patients’ reception of AI, mistrust, or apprehension is a key factor that needs to be taken into consideration [55,56].

## 7. Discussion

The important technological advancement of multimodal capabilities places the medical world at a critical intersection regarding where the medical profession is headed, and what will be the role of AI in the process of patient care. It is clear that AI multimodal technology has the potential to revolutionize clinical care, and it is already implemented in various medical fields, either as an additive tool for physicians or by performing complete tasks independently.

This also raises further moral and philosophical questions, regarding the perceived importance of the simple human touch in medical care [57]. Human touch is an essential part of human interaction; it is at the core of social, cultural, and professional relationships, and it is an integral part of clinical care [58]. The integration of multimodal technology in healthcare is already taking place in hospitals and administrative systems worldwide. However, careful attention must be given to its impact on clinical care, not only in the medical sense but also in terms of its emotional, psychological, and social impacts on patients in the clinical setting.

Several challenges mentioned in the studies that are important to address are bias and liability. Bias in AI models trained on unrepresentative or skewed data could lead to inaccurate or discriminatory outcomes, especially for under-represented populations. This can exacerbate healthcare disparities, and further cause mistrust in AI [59]. Liability concerns revolve around accountability. If AI makes incorrect decisions, such as misdiagnoses or flawed treatment recommendations, determining whether the responsibility lies with the healthcare provider, the developer, or the AI system itself presents complex legal and ethical dilemmas, complicating the widespread adoption of AI in healthcare.

An important aspect of AI integration in healthcare and clinical practice is patients’ perception and opinion on AI technology. While AI has the potential to enhance diagnostic accuracy and personalize treatment plans, patients’ trust in these systems remains a critical factor [60]. While some patients appreciate the efficiency and precision AI can bring, others might express concerns over reduced human interaction, fearing that AI-driven healthcare may feel impersonal or lack the empathy inherent in human clinicians. These apprehensions are often more pronounced in older adults or individuals from culturally diverse backgrounds who may be less familiar with advanced technologies or more reliant on traditional doctor–patient relationships [61].

Furthermore, AI systems may inadvertently exacerbate healthcare disparities in resource-limited populations. AI models are often trained on datasets that do not adequately represent marginalized or low-income groups, leading to biases in diagnosis or treatment recommendations that could disproportionately affect these populations [62]. In resource-limited settings, the high cost of implementing AI technologies may also restrict access, potentially creating a digital divide where wealthier institutions and patients benefit from AI advancements while under-resourced communities are left behind [63]. Thus, while AI integration offers promising advancements in medical practice, it must be carefully implemented with consideration of patient perspectives, cultural sensitivities, and equitable access to avoid widening existing healthcare disparities.

Our perspective is intended to provide a broad overview of multimodal AI in clinical practice (Table 2). However, future works should systematically review the literature, using methodologies such as PRISMA, and explore meta-analyses of quantitative results. Additionally, structured interviews with healthcare professionals and patients could provide diverse insights into the real-world implementation of multimodal AI, helping to assess both its benefits and limitations in clinical environments. Additionally, future research should also focus on comparison studies between multimodal AI and traditional diagnostic methods. Long-term-follow-up, original studies evaluating patient outcomes, and assessments of cost-effectiveness across different healthcare settings, will be critical to understanding the broader implications of AI technologies in clinical environments.

Lastly, our perspective is intended to provide a broad overview of multimodal AI in clinical practice. However, future works should also systematically review the literature, using methodologies such as PRISMA, and explore meta-analyses of quantitative results.

## 8. Conclusions

Multimodal AI applications in clinical practice integrate diverse data sources such as medical images, electronic health records (EHRs), sound recordings, and genomic data to enhance diagnostic accuracy, treatment planning, and patient monitoring. By combining multiple data modalities, AI systems provide a comprehensive view of patient health, potentially improving diagnosis and treatment. In several studies we reviewed, the application of AI increased efficiency, reduced waiting times, and proved to be a valuable tool in the hands of proficient healthcare staff.

The further application of AI multimodal technology can also be expanded and used in the setting of medical education, where AI models assist medical students and physicians prepare for exams, practice clinical scenarios, and enhance learning. However, such a rapid development of technology is not without its challenges, such as data integration, algorithm transparency, bias in data, hallucinations, and ensuring equitable outcomes across diverse and disadvantaged populations. Additionally, the accountability and liability of AI-driven decisions in complex healthcare environments pose significant ethical and legal concerns.

These applications show great potential in enhancing clinical practice and creating a more calculated and holistic approach to healthcare, but due to its many limitations, this technology remains as a tool, its applicability is still heavily user-dependent, and it requires careful oversight by experienced practitioners.

## Figures and Tables

**Table 1:** Some examples of current AI multimodal platforms.

AI Platform	Capabilities	Input Modalities	Typical Uses
GPT-4 Vision [18] (OpenAI)	Text and image generation, comprehension, translation, summarization	Text, image, video	Content creation, conversation, coding assistance, data analysis, education, graphic design
DALL-E [19] (OpenAI)	Image generation from textual descriptions	Text	Graphic design, art creation, visual content generation, advertising
CLIP [20] (OpenAI)	Understanding and classifying images in the context of natural language	Text, image	Image search, analysis, classification based on textual descriptions
Whisper [21] (OpenAI)	Speech-to-text transcription, translation	Audio	Transcription services, language translation of spoken content, accessibility tools
CoPilot [22] (GitHub)	Code generation and suggestion based on natural language	Text	Software development assistance, debugging, code review, educational tools
Gemini [23] (Google)	Text and image generation, comprehension, translation, summarization	Text, image, video	Conversational agents, customer service bots, personal assistants, interactive storytelling, education
DeepMind’s Perceiver [24]	Processing and integrating different types of data	Text, image, audio, video	Universal data processing, cross-modal information retrieval, games, simulations, research
Midjourney [25]	Image generation based on textual prompts	Text	Visual storytelling, concept art, design exploration
Stable Diffusion [26]	Text-to-image generation, image editing	Text, image	Content creation, digital art, image editing, marketing
Meta.AI Llama [27]	Text and image generation, comprehension, translation, summarization	Text, image	Content creation, conversational interfaces, data analysis, educational tools

**Table 2:** Comparison and summarization of the strengths and weaknesses of various AI-based multimodal tools across the different studies mentioned.

Field	Study	Strengths	Weaknesses
Radiology	Zhu et al., 2024 (*Int J Surg*) [28]	Strong in interpreting radiological images, handles large image–text datasets.	Limited medical domain expertise; potential for misinterpretation of complex cases.
Mental health	Elyoseph et al., 2024 (*JMIR Ment Health*) [29]	Effective at recognizing emotions from visual and textual data, helpful in mental health applications.	Potential for biases in emotion recognition and difficulty with more nuanced emotional contexts.
Neuroimaging	Biessmann et al., 2011 (*IEEE Rev Biomed Eng*) [30]	Robust integration of different neuroimaging modalities; enhances understanding of brain function.	Requires significant computational resources and expertise; challenges with data standardization.
Radiology	Brin et al., 2024 (*Eur Radiol*) [31]	Accurate interpretation of radiological images, effective across diverse clinical cases.	Performance varies with image complexity; potential for hallucination of results in complex imaging cases.
Ophthalmology	Sorin et al., 2024 (*MedRxiv*) [36]	Analysis of external ocular images with or without clinical context.	Performance was inferior to non-ophthalmologist physicians, and was only evaluated based on external images versus OCT or fundoscopy.
Pulmonary	Cahan et al., 2023 (*Sci Rep*) [32]	Improves mortality prediction by fusing clinical and imaging data, supports personalized treatment planning.	Model complexity and interpretability challenges; requires large, high-quality datasets.
Biomedicine	Acosta et al., 2022 (*Nat Med*) [33]	Integrates diverse medical data (imaging, clinical) for diagnostics, facilitates personalized care.	High dependency on comprehensive, high-quality datasets; concerns about generalizability in clinical practice.
Virtual reality in healthcare	Kouijzer et al., 2023 (*Implement Sci Commun*) [34]	Enhances patient engagement, effective for rehabilitation and training.	Implementation challenges, especially in integrating VR with existing healthcare systems.
Neurology	Ramanarayanan, 2024 (*J Speech Lang Hear Res*) [35]	Effective for remote monitoring and assessments, supports telehealth initiatives.	Privacy concerns and limited accuracy for certain complex conditions.
Immunology	Indolfi et al., 2024 (*Front Med*) [37]	Supports continuity of care from childhood to adulthood, personalized treatment recommendations.	Limited data on long-term outcomes; potential for biases in decision making.
Operations and patient management	Zhu et al., 2022 (*Front Surg*) [38]	Improves operational efficiency, integrates multimodal ID technology for patient and material management.	Complex integration with existing hospital systems; steep learning curve for users.
Radiology	Mohsen et al., 2022 (*Sci Rep*) [40]	Effective in combining EHR and imaging data for enhanced decision making, predictive analytics capabilities.	Requires high-quality, standardized datasets; concerns about patient privacy and data security.
Oncology	Lipkova et al., 2022 (*Cancer Cell*) [41]	Enables comprehensive analysis of multimodal cancer data (genomics, imaging), supports precision oncology.	Computationally expensive; challenges in scaling for real-time clinical applications.
Oncology/genomics	Shao et al., 2023 (*Semin Cancer Biol*) [42]	Enhances precision in predicting gene mutations through multimodal integration of genomics and clinical data.	Model complexity may hinder clinical interpretability; requires extensive training datasets.
Mental health	Alhuwaydi, 2024 (*Risk Manag Healthc Policy*) [43]	Improves accessibility to mental healthcare, supports early diagnosis and intervention through multimodal data.	Ethical concerns, potential biases in AI-driven mental health interventions, and lack of human empathy.
Mental health	Ettman & Galea, 2023 (*JMIR Ment Health*) [48]	Enhances population mental health monitoring, addresses large-scale public health issues with AI interventions.	Risks of widening socioeconomic disparities through AI implementation; data privacy concerns.

## Data Availability

No new data were created or analyzed in this study. Data sharing is not applicable to this article.

## References

[1] Yapijakis C. (2009). Hippocrates of Kos, the father of clinical medicine, and Asclepiades of Bithynia, the father of molecular medicine. Review. In Vivo.

[2] Donoso F.A., Arriagada S.D. (2020). René Théophile Hyacinthe Laënnec (1781–1826). Two hundred years of the stethoscope. A brief overview. René Théophile Hyacinthe Laënnec (1781–1826). Doscientos años del uso del estetoscopio. Una breve reseña. Arch. Argent Pediatr..

[3] Bruhn J.G. (1978). The doctor’s touch: Tactile communication in the doctor-patient relationship. South Med. J..

[4] Bijland L.R., Bomers M.K., Smulders Y.M. (2013). Smelling the diagnosis: A review on the use of scent in diagnosing disease. Neth. J. Med..

[5] Eknoyan G., Nagy J. (2005). A history of diabetes mellitus or how a disease of the kidneys evolved into a kidney disease. Adv. Chronic Kidney Dis..

[6] Heath C., Luff P., Svensson M.S. (2003). Technology and medical practice. Sociol. Health Illn..

[7] Luo L., Luo Q., Tang L. (2020). Diagnostic value and clinical significance of MRI and CT in detecting lymph node metastasis of early cervical cancer. Oncol. Lett..

[8] Glicksberg B.S., Johnson K.W., Dudley J.T. (2018). The next generation of precision medicine: Observational studies, electronic health records, biobanks and continuous monitoring. Hum. Mol. Genet..

[9] Taha A.R., Shehadeh M., Alshehhi A., Altamimi T., Housser E., Simsekler M.C., Alfalasi B., Al Memari S., Al Hosani F., Al Zaabi Y. (2022). The integration of mHealth technologies in telemedicine during the COVID-19 era: A cross-sectional study. PLoS ONE.

[10] Soffer S., Ben-Cohen A., Shimon O., Amitai M.M., Greenspan H., Klang E. (2019). Convolutional Neural Networks for Radiologic Images: A Radiologist’s Guide. Radiology.

[11] Klang E. (2018). Deep learning and medical imaging. J. Thorac. Dis..

[12] Vaswani A., Shazeer N., Parmar N., Uszkoreit J., Jones L., Gomez A.N., Kaiser L., Polosukhin I. (2017). Attention Is All You Need. arXiv.

[13] Zhang Y., Liu C., Liu M., Liu T., Lin H., Huang C.B., Ning L. (2023). Attention is all you need: Utilizing attention in AI-enabled drug discovery. Brief Bioinform..

[14] Sorin V., Klang E. (2023). Large language models and the emergence phenomena. Eur. J. Radiol. Open.

[15] Azad R., Kazerouni A., Heidari M., Aghdam E.K., Molaei A., Jia Y., Jose A., Roy R., Merhof D. (2024). Advances in medical image analysis with vision Transformers: A comprehensive review. Med. Image Anal..

[16] Meskó B. (2023). The Impact of Multimodal Large Language Models on Health Care’s Future. J. Med. Internet Res..

[17] Ma J., Fong S.H., Luo Y., Bakkenist C.J., Shen J.P., Mourragui S., Wessels L.F.A., Hafner M., Sharan R., Peng J. (2021). Few-shot learning creates predictive models of drug response that translate from high-throughput screens to individual patients. Nat. Cancer.

[18] Zhou Y., Ong H., Kennedy P., Wu C.C., Kazam J., Hentel K., Flanders A., Shih G., Peng Y. (2024). Evaluating GPT-V4 (GPT-4 with Vision) on Detection of Radiologic Findings on Chest Radiographs. Radiology.

[19] Zhu L., Mou W., Wu K., Zhang J., Luo P. (2024). Can DALL-E 3 Reliably Generate 12-Lead ECGs and Teaching Illustrations?. Cureus.

[20] Eslami S., de Melo G., Meinel C. (2021). Does CLIP Benefit Visual Question Answering in the Medical Domain as Much as it Does in the General Domain?. arXiv.

[21] Graham C., Roll N. (2024). Evaluating OpenAI’s Whisper ASR: Performance analysis across diverse accents and speaker traits. JASA Express Lett..

[22] Lu M.Y., Chen B., Williamson D.F.K., Chen R.J., Zhao M., Chow A.K., Ikemura K., Kim A., Pouli D., Patel A. (2024). A multimodal generative AI copilot for human pathology. Nature.

[23] Masalkhi M., Ong J., Waisberg E., Lee A.G. (2024). Google DeepMind’s gemini AI versus ChatGPT: A comparative analysis in ophthalmology. Eye.

[24] Jaegle A., Gimeno F., Brock A., Zisserman A., Vinyals O., Carreira J. (2021). Perceiver: General Perception with Iterative Attention. arXiv.

[25] Buzzaccarini G., Degliuomini R.S., Borin M., Fidanza A., Salmeri N., Schiraldi L., Di Summa P.G., Vercesi F., Vanni V.S., Candiani M. (2024). The Promise and Pitfalls of AI-Generated Anatomical Images: Evaluating Midjourney for Aesthetic Surgery Applications. Aesthetic Plast. Surg..

[26] Dehouche N., Dehouche K. (2023). What’s in a text-to-image prompt? The potential of stable diffusion in visual arts education. Heliyon.

[27] Li Y., Li Z., Zhang K., Dan R., Jiang S., Zhang Y. (2023). ChatDoctor: A Medical Chat Model Fine-Tuned on a Large Language Model Meta-AI (LLaMA) Using Medical Domain Knowledge. Cureus.

[28] Zhu L., Mou W., Lai Y., Chen J., Lin S., Xu L., Lin J., Guo Z., Yang T., Lin A. (2024). Step into the era of large multimodal models: A pilot study on ChatGPT-4V(ision)’s ability to interpret radiological images. Int. J. Surg. (Lond. Engl.).

[29] Elyoseph Z., Refoua E., Asraf K., Lvovsky M., Shimoni Y., Hadar-Shoval D. (2024). Capacity of Generative AI to Interpret Human Emotions from Visual and Textual Data: Pilot Evaluation Study. JMIR Ment. Health.

[30] Biessmann F., Plis S., Meinecke F.C., Eichele T., Müller K.R. (2011). Analysis of multimodal neuroimaging data. IEEE Rev. Biomed. Eng..

[31] Brin D., Sorin V., Barash Y., Konen E., Glicksberg B.S., Nadkarni G.N., Klang E. (2024). Assessing GPT-4 multimodal performance in radiological image analysis. Eur. Radiol..

[32] Cahan N., Klang E., Marom E.M., Soffer S., Barash Y., Burshtein E., Konen E., Greenspan H. (2023). Multimodal fusion models for pulmonary embolism mortality prediction. Sci. Rep..

[33] Acosta J.N., Falcone G.J., Rajpurkar P., Topol E.J. (2022). Multimodal biomedical AI. Nat Med..

[34] Kouijzer M.M.T.E., Kip H., Bouman Y.H.A., Kelders S.M. (2023). Implementation of virtual reality in healthcare: A scoping review on the implementation process of virtual reality in various healthcare settings. Implement. Sci. Commun..

[35] Ramanarayanan V. (2024). Multimodal Technologies for Remote Assessment of Neurological and Mental Health. J. Speech Lang. Hear. Res. JSLHR.

[36] Sorin V., Kapelushnik N., Hecht I., Zloto O., Glicksberg B.S., Bufman H., Barash Y., Nadkarni G.N., Klang E. (2023). GPT-4 Multimodal analysis on ophthalmology clinical cases including text and images. medRxiv.

[37] Indolfi C., Klain A., Dinardo G., Decimo F., Miraglia Del Giudice M. (2024). Artificial intelligence in the transition of allergy: A valuable tool from childhood to adulthood. Front. Med..

[38] Zhu Y., Sun X., Huang Y., Song X., Liu L., Feng L., Zhang Y. (2022). Application of multimodal identification technology in the innovative management operation department. Front. Surg..

[39] Kwon YJ F., Toussie D., Finkelstein M., Cedillo M.A., Maron S.Z., Manna S., Voutsinas N., Eber C., Jacobi A., Bernheim A. (2020). Combining Initial Radiographs and Clinical Variables Improves Deep Learning Prognostication in Patients with COVID-19 from the Emergency Department. Radiol. Artif. Intell..

[40] Mohsen F., Ali H., Hajj N.E., Shah Z. (2022). Artificial intelligence-based methods for fusion of electronic health records and imaging data. Sci. Rep..

[41] Lipkova J., Chen R.J., Chen B., Lu M.Y., Barbieri M., Shao D., Vaidya A.J., Chen C., Zhuang L., Williamson D.F.K. (2022). Artificial intelligence for multimodal data integration in oncology. Cancer Cell.

[42] Shao J., Ma J., Zhang Q., Li W., Wang C. (2023). Predicting gene mutation status via artificial intelligence technologies based on multimodal integration (MMI) to advance precision oncology. Semin. Cancer Biol..

[43] Alhuwaydi A.M. (2024). Exploring the Role of Artificial Intelligence in Mental Healthcare: Current Trends and Future Directions-A Narrative Review for a Comprehensive Insight. Risk Manag. Healthc. Policy.

[44] Dott C., Mamarelis G., Karam E., Bhan K., Akhtar K. (2022). Emotional Intelligence and Good Medical Practice: Is There a Relationship?. Cureus.

[45] Flores L., Kim S., Young S.D. (2024). Addressing bias in artificial intelligence for public health surveillance. J. Med. Ethics.

[46] Sorin V., Soffer S., Glicksberg B.S., Barash Y., Konen E., Klang E. (2023). Adversarial attacks in radiology—A systematic review. Eur. J. Radiol..

[47] Gopalkrishnan N. (2018). Cultural Diversity and Mental Health: Considerations for Policy and Practice. Front. Public Health.

[48] Ettman C.K., Galea S. (2023). The Potential Influence of AI on Population Mental Health. JMIR Ment. Health.

[49] Kung T.H., Cheatham M., Medenilla A., Sillos C., De Leon L., Elepaño C., Madriaga M., Aggabao R., Diaz-Candido G., Maningo J. (2023). Performance of ChatGPT on USMLE: Potential for AI-assisted medical education using large language models. PLoS Digit. Health.

[50] Artsi Y., Sorin V., Konen E., Glicksberg B.S., Nadkarni G., Klang E. (2024). Large language models for generating medical examinations: Systematic review. BMC Med. Educ..

[51] Samhammer D., Roller R., Hummel P., Osmanodja B., Burchardt A., Mayrdorfer M., Duettmann W., Dabrock P. (2022). “Nothing works without the doctor:” Physicians’ perception of clinical decision-making and artificial intelligence. Front. Med..

[52] Poon A.I.F., Sung J.J.Y. (2021). Opening the black box of AI-Medicine. J. Gastroenterol. Hepatol..

[53] Celi L.A., Cellini J., Charpignon M., Dee E.C., Dernoncourt F., Eber R., Mitchell W.G., Moukheiber L., Schirmer J., Situ J. (2022). Sources of bias in artificial intelligence that perpetuate healthcare disparities—A global review. PLoS Digit. Health.

[54] Athaluri S.A., Manthena S.V., Kesapragada V.K., Yarlagadda V., Dave T., Duddumpudi R.T. (2023). Exploring the Boundaries of Reality: Investigating the Phenomenon of Artificial Intelligence Hallucination in Scientific Writing Through ChatGPT References. Cureus.

[55] Esmaeilzadeh P., Mirzaei T., Dharanikota S. (2021). Patients’ Perceptions Toward Human-Artificial Intelligence Interaction in Health Care: Experimental Study. J. Med. Internet Res..

[56] Robertson C., Woods A., Bergstrand K., Findley J., Balser C., Slepian M.J. (2023). Diverse patients’ attitudes towards Artificial Intelligence (AI) in diagnosis. PLoS Digit. Health.

[57] Pepito J.A.T., Babate F.J.G., Dator W.L.T. (2023). The nurses’ touch: An irreplaceable component of caring. Nurs. Open.

[58] Nazer L.H., Zatarah R., Waldrip S., Ke J.X.C., Moukheiber M., Khanna A.K., Hicklen R.S., Moukheiber L., Moukheiber D., Ma H. (2023). Bias in artificial intelligence algorithms and recommendations for mitigation. PLoS Digit. Health.

[59] Bottomley D., Thaldar D. (2023). Liability for harm caused by AI in healthcare: An overview of the core legal concepts. Front Pharmacol..

[60] Steerling E., Siira E., Nilsen P., Svedberg P., Nygren J. (2023). Implementing AI in healthcare-the relevance of trust: A scoping review. Front Health Serv..

[61] Zhang M. (2023). Older people’s attitudes towards emerging technologies: A systematic literature review. Public Underst. Sci..

[62] D’Elia A., Gabbay M., Rodgers S., Kierans C., Jones E., Durrani I., Thomas A., Frith L. (2022). Artificial intelligence and health inequities in primary care: A systematic scoping review and framework. Fam. Med. Community Health.

[63] Alami H., Rivard L., Lehoux P., Hoffman S.J., Cadeddu S.B.M., Savoldelli M., Samri M.A., Ag Ahmed M.A., Fleet R., Fortin J. (2020). Artificial intelligence in health care: Laying the Foundation for Responsible, sustainable, and inclusive innovation in low- and middle-income countries. Glob. Health.

